# Hyperspectral imaging with machine learning for in vivo skin carcinoma margin assessment: a preliminary study

**DOI:** 10.1007/s13246-024-01435-8

**Published:** 2024-05-21

**Authors:** Sorin Viorel Parasca, Mihaela Antonina Calin, Dragos Manea, Roxana Radvan

**Affiliations:** 1 Carol Davila University of Medicine and Pharmacy, 37 Dionisie Lupu Street, Bucharest, Romania; 2 Emergency Clinical Hospital for Plastic, Reconstructive Surgery and Burns, 218 Grivitei Street, Bucharest, Romania; 3 National Institute of Research and Development for Optoelectronics– INOE 2000, 409 Atomistilor Street, 077125 Magurele, Ilfov, P.O. BOX MG5, Romania

**Keywords:** Basal cell carcinoma, Squamous cell carcinoma, Segmentation, Spectral Angle Mapper, Classification maps, Tumor margins

## Abstract

Surgical excision is the most effective treatment of skin carcinomas (basal cell carcinoma or squamous cell carcinoma). Preoperative assessment of tumoral margins plays a decisive role for a successful result. The aim of this work was to evaluate the possibility that hyperspectral imaging could become a valuable tool in solving this problem. Hyperspectral images of 11 histologically diagnosed carcinomas (six basal cell carcinomas and five squamous cell carcinomas) were acquired prior clinical evaluation and surgical excision. The hyperspectral data were then analyzed using a newly developed method for delineating skin cancer tumor margins. This proposed method is based on a segmentation process of the hyperspectral images into regions with similar spectral and spatial features, followed by a machine learning-based data classification process resulting in the generation of classification maps illustrating tumor margins. The Spectral Angle Mapper classifier was used in the data classification process using approximately 37% of the segments as the training sample, the rest being used for testing. The receiver operating characteristic was used as the method for evaluating the performance of the proposed method and the area under the curve as a metric. The results revealed that the performance of the method was very good, with median AUC values of 0.8014 for SCCs, 0.8924 for BCCs, and 0.8930 for normal skin. With AUC values above 0.89 for all types of tissue, the method was considered to have performed very well. In conclusion, hyperspectral imaging can become an objective aid in the preoperative evaluation of carcinoma margins.

## Introduction

The incidence of skin cancers (basal cell carcinoma (BCC), squamous cell carcinoma (SCC) and melanoma) has been increasing for many years and there is a constant need for more precise treatment options which can completely eradicate the malignancy and reduce morbidity and mortality. Current treatment options for skin cancers include cryotherapy [1], surgery [2], radiotherapy [3], and immunotherapy [4]. Although significant progress has been made in each of these treatment options, surgery is still considered the gold standard for skin cancer treatment [5]. However, the determination of the tumoral margin is a key factor in the treatment management, both in terms of the treatment effectiveness (complete removal of the tumor and reducing the possibility of recurrence), as well as the minimizing of the functional and esthetic downfalls associated with an extensive excision.

Commonly used methods for intraoperative tumor margin assessment involve visual examination and palpation by a surgeon combined with frozen section analysis or Mohs surgery [6]. However, both modalities have some drawbacks. Visual assessment is subjective and highly dependent on the surgeon’s experience. The frozen section analysis and Mohs technique can be affected by some errors that may occur during the sampling and interpretation [7], being otherwise laborious and time-consuming, leading to the longer operative time. Therefore, there is a great need for new and improved techniques that allow a clearer delineation of tumor margins as an aid to make correct decisions about the extent of surgical excision. Some optical methods have so far demonstrated their merits in this area, such as: dermoscopy [8], reflectance confocal microscopy [9], Raman spectroscopy [10], diffuse reflectance spectroscopy [11], photoacoustic tomography [12], and optical coherence tomography [13]. Recently, hyperspectral imaging (HSI) has been shown to be a possible new non-invasive option for tumor margins assessment. Hyperspectral imaging is an imaging technique that, by combining conventional imaging with spectroscopy, has the advantage of simultaneously providing both spatial and spectral information that is correlated with the chemical composition and structure of the investigated biological tissues. Such spectral and spatial information obtained from hundreds of narrow, contiguous spectral bands at high spectral resolution has been exploited by several researcher groups to assess tumor margins in both *ex-vivo* and in vivo studies with promising results. Thus, Fei et al. [14] developed a label-free hyperspectral imaging method for tumor margin assessment in surgical tissue specimens of head and neck cancers that was shown to have great potential for oral cavity tumor margin assessment (average accuracy: 90 ± 8%, sensitivity: 89 ± 9%, and specificity:91 ± 6%) and thyroid (average accuracy: 94 ± 6%, sensitivity: 94 ± 6%, and specificity:95 ± 6%). Halicek et al. [15] reported in 2019 the first study on the use of reflectance-based hyperspectral imaging combined with a convolutional neural network for cancer margin detection on a large dataset of head and neck squamous cell carcinoma (293 tissue specimens) and demonstrated that the cancer margin in *ex-vivo* specimens can be accurately detected (AUCs > 0.80–0.90) in less than 2 min. A pixel-level tumor margin assessment method on surgical specimens of head and neck squamous cell carcinoma based on hyperspectral imaging combined with a fully convolutional network model based on U-Net architecture was also propose by Ma et al. [16] with promising results for fast tumor detection (several minutes) and high-precision margin assessment (AUC of 0.88) during surgery. More recently, Pertzborn et al. [17] implemented a combined method based on hyperspectral imaging and machine learning as a new tool for accurate intraoperative assessment of the margin of oral squamous cell carcinoma in fresh-frozen unstained tissue slices (accuracy: 0.76, specificity: 0.89 and sensitivity: 0.48). Aloupogianni et al. [18] reported the use of hyperspectral imaging to investigate tumor margin detection on pigmented skin lesions during gross pathology with good performance for melanocytic lesions, but not for margins detection in some cases of basal cell carcinoma. Encouraging results have also been reported on *ex-vivo* applications of hyperspectral imaging in breast cancer margin assessment [19, 20].

Unlike ex vivo studies that are more numerous and cover a wider range of cancerous tumors, to our knowledge, only three in vivo studies have so far demonstrated the usefulness of hyperspectral imaging in preoperative delineation of tumor margins. Thus, Neittaanmäki-Perttu et al [21] reported on the ability of hyperspectral imaging to detect in vivo subclinical margins of lentigo maligna and lentigo maligna melanoma. A new non-invasive method based on hyperspectral imaging and diffusion maps technique was proposed by Zheludev et al. [22] in 2015, with demonstrated performance in delineation of the same types of cancer, namely lentigo maligna and lentigo maligna melanoma. More recently, Salmivuori et al. [23] reported the first study on the in vivo delineation of BCC margins in the head and neck area using hyperspectral imaging. Their results revealed that hyperspectral imaging can easily and fast delineate ill-defined basal cell carcinoma. In all these in vivo studies, tumor margins determined by hyperspectral imaging were compared to those assessed clinically by the physician and confirmed by histological results. The in vivo studies were performed however, only for a small number of tumor types (mostly pigmentary tumors: lentigo maligna, lentigo maligna melanoma and basal cell carcinoma) and a limited number of discrete locations (faces, scalps, head and neck) without validation from other standard method such as dermoscopy.”

The aim of this study was to propose a new non-invasive in vivo approach for delineating the margins of skin carcinomas (BCC and SCC). This new approach combines segmentation and classification of hyperspectral images of skin carcinomas and surrounding normal skin in order to facilitate the integrative use of spectral and spatial zonal features to identify the group regions (normal skin and tumor) with similar spectral properties and providing segmentation statistics, characterizing their overall shapes, and locating their boundaries for accurate data classification. Our method provides an automated tool for assessing skin cancer margins which can help the physicians in choosing the excision extent. All previous in vivo studies used pixel-based classification methods to delineate tumor margins. Our approach proposes a region-based classification method as a new and more precise way of preoperative delineation of tumor margins.

To fulfill this main objective the following issues were addressed: (1) establishing the experimental conditions for hyperspectral images acquisition of different skin carcinomas; (2) identification of hyperspectral image processing methods to eliminate noise and reduce data dimensionality; (3) applying segmentation algorithms to create regions with similar features; (4) hyperspectral data analysis using spectral classifiers for segments classification and tumor margin mapping, and (5) evaluation of the effectiveness of the proposed approach in the delineation of the tumor margin using the receiver operating characteristic curve (ROC) and comparison with the clinical examination, histopathology, and dermoscopy.

## Materials and methods

### Patients

Eleven patients, 8 women and 3 men, aged between 55 and 88 years (mean age ± standard deviation: 77.82± 9.93 years) with skin carcinomas (6 BCC and 5 SCC) of the face or scalp admitted to the Emergency Clinical Hospital for Plastic, Reconstructive Surgery and Burns, Bucharest, between January and March 2023, were selected for this preliminary study (Table 1). Inclusion criteria were clinically diagnosed skin tumors and histologically confirmed as BCC and SCC.

**Table 1:** Clinical and histopathological data of the patients with BCCs and SCCs included in the study group

Patient	Age (years)/sex	Tumor location	Histopathological type
1	82/F	Right temporal region	Nodular BCC
2	82/F	Left cheek	Nodular BCC
3	65/M	Upper lip	Nodular and infiltrative micronodular BCC
4	88/M	Left temporal region	Superficial and nodular BCC
5	88/M	Right temporal region	Moderate differentiated SCC
6	55/F	Right malar area	Moderate differentiated SCC
7	81/F	Nasolabial region	Moderate differentiated SCC
8	81/F	Right temporal region	Well diferentiated SCC
9	80/F	Left submandibular region	Bowen’s disease
10	73/F	Left nasal ala and cheek region	Nodular and infiltrative BCC
11	81/F	Left temporal region	Infiltrative BCC

All patients gave informed consent prior to participating in this study. The procedures performed in this study involving human participants followed the 1964 Helsinki declaration and its later amendments and were in accordance with the ethical standards of the Emergency Clinical Hospital for Plastic, Reconstructive Surgery and Burns Research Committee.

### Clinical examination

The patients were clinically examined by two independent physicians after the hyperspectral images acquisition, and clinical margins were established using surgical loupes (2.5X).

### Histological evaluation

All skin tumors were excised after acquisition of hyperspectral images of the tumors and surrounding skin, and the resulting surgical specimens were collected and transported in formalin to the pathology department for processing and histopathological analysis by the pathologist. Histopathological diagnoses received later were: 6 BCC and 5 SCC (Table 1). Histological results were used to confirm the type of skin carcinoma and the presence or absence of residual tumor.

### Dermoscopic evaluation

All skin carcinomas were examined with a dermoscope (Heine Delta 30, Heine Optotechnik GmbH & Co. KG, Gilching, Germany). The dermoscopic locations of tumor margins were evaluated and analyzed by two dermatologists and the distance from the tumor center to its farthest margin (D_max−dermo_) was measured for comparison. For more accurate data analysis, regions of interest relative only to the tumor margin and the surrounding normal skin, confirmed by pathology, were chosen in each image.

### Hyperspectral images acquisition and processing

Hyperspectral images of the tumor areas were acquired using a hyperspectral imaging system based on an imaging spectrograph (ImSpector V8E, Specim, Oulu, Finland) coupled to a charge-coupled device (DX4 camera, Kappa, Gleichen, Germany). This system allows the acquisition of images (205 spectral bands) in the spectral range (400–800) nm at a spatial resolution of 348 × 260 pixels and spectral resolution of 1.97 nm. Scanning of the investigated tumor area was provided by a single-axis galvanometer scanning mirror system (GVS211, Thorlabs, New Jersey, USA) equipped with a broadband dielectric mirror (average reflectance > 95% in the spectral domain (400–750) nm). The illumination system uses two 300 W halogen lamps (OSRAM, Munich, Germany), equipped with diffusion filters (Kaiser Fototechnik GmbH and Co. KG, Buchen, Germany) mounted on either side of the investigated tumor area at an angle of approximately 45º to ensure a uniform illumination of the investigated area. Acquisition and storage of hyperspectral data in a computer was performed using SpectralDAQ software (Specim, Oulu, Finland). Pre-processing and analysis of the stored hyperspectral data was performed with ENVI data processing and analysis software v.5.1 (Exelis Visual Information Solutions, Boulder, Colorado, USA).

The hyperspectral data captured by this hyperspectral imaging system is expressed by a digital number (DN) and in order to extract relevant information (both spectral and spatial) from such data, the pixel value must be converted to reflectance. Moreover, hyperspectral images may contain some non-informative background or data (elements present in the scene other than the tumor area to be investigated). The presence of these two factors can considerably affect the subsequent image analysis. Their influence was reduced by a two-step processing of the hyperspectral images: (1) calibration of hyperspectral images with black and white reference images and (2) manual selection of a region of interest (ROI) related only to the tumor area without background elements or other artifacts.

**Fig. 1:**
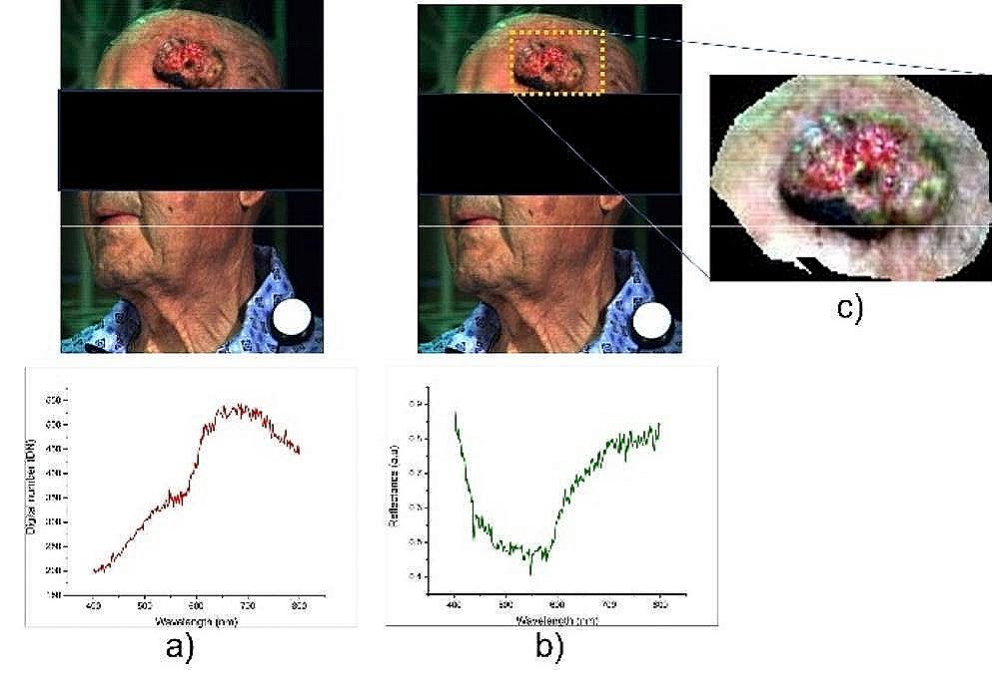
Images of squamous cell carcinoma of the temporal region. (**a**) original hyperspectral image (I_O_) (pixel values are expressed in digital number (DN)); (**b**) calibrated hyperspectral image (I_cal_) (pixel values are expressed in reflectance (R)); (**c**) region of interest (ROI)

Calibration of the hyperspectral images of the investigated tumor areas was performed using as a dark reference image (D_ref_) an image obtained by completely covering the lens of the imaging spectrograph with its black cap and turning off the illumination system and as a white reference image (W_ref_), the image of a polytetrafluoroethylene (PTFE) reference tile (model WS-2, Avantes, Apeldoorn, The Netherlands) with ∼98% reflectance in the VIS-NIR spectral range, located in the investigated scene near the tumor area (Fig. 1). The calibrated image (I_cal_) was obtained using Eq. (1):

1 $$ {I}_{cal}=\frac{{I}_{o}-{D}_{ref}}{{W}_{ref}-{D}_{ref}}$$

where: I_cal_ is the calibrated hyperspectral image of the tumor area, in units of reflectance, I_O_ is the original hyperspectral image of the tumor area, D_ref_ is the dark reference image, and W_ref_ is the white reference image. The calibrated images were used as bases for subsequent analysis.

Data dimensionality reduction, removal of irrelevant information from hyperspectral images and data simplification to the maximum extent were performed in the second step by selecting a region of interest (ROI) containing only the tumor area and surrounding normal skin (Fig. 1c) so that the hyperspectral data was transformed into a small dataset to be used for the subsequent analysis.

### Non-invasive method for delineating the margins of skin carcinomas (TMD)

**Fig. 2:**
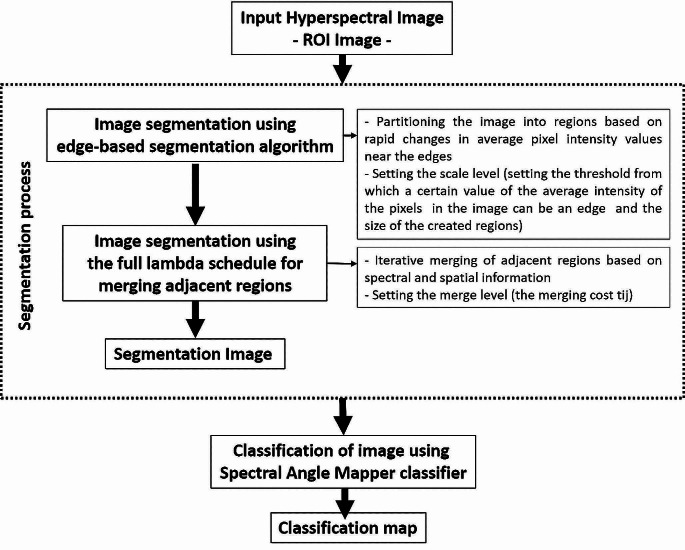
Flowchart of the proposed TMD method for the delimitation of the margins of skin carcinomas

In this study, a method (TMD) is proposed to detect the margins of skin cancer tumors in hyperspectral images. The proposal is based on two assumptions: (a) pixels belonging to a certain region can be classified into a class and (b) neighboring pixels are highly correlated [24]. Based on these assumptions, a hyperspectral image segmentation process was first performed in order to identify and group regions with similar spectral properties, followed by a classification process of these regions. These processes were implemented in the proposed TMD method in three main steps (Fig. 2):

The first step consists of performing segmentation of hyperspectral images using an edge-based segmentation algorithm implemented by ENVI v.5.1 software [25], which extracts boundaries and creates regions based on rapid changes in average pixel intensity. To avoid over-segmentation, in the second step, the full lambda-schedule algorithm introduced by Robinson et al. [26] was used to combine small areas into larger areas with similar features. This image segmentation algorithm iteratively merges adjacent pairs of neighboring segments (Si, Sj) based on their spectral and spatial features and selects the best pair to merge for which the merging cost t_ij_ is less than a defined threshold T value (Eq. 2):

2 $$ {t}_{ij}=\frac{\frac{\left|{S}_{i}\right|\left|{S}_{j}\right|}{\left|{S}_{i}\right|+\left|{S}_{j}\right|}{||{v}_{i}-{v}_{j}||}^{2}}{l\left(\partial \left({S}_{i,}{S}_{j}\right)\right)}<T$$

where: t_ij_ is the merging cost, S_i_ and S_j_ are segments i and j respectively of the image,|S_i_| and|S_j_| are the areas of segments i and j, respectively, v_i_ is the average value in segment i, v_j_ is the average value in segment j, $$||{v}_{i}-{v}_{j}|| $$ is the Euclidean distance between the spectral values of S_i_ and S_j_ segments, and $$ l\left(\partial \left({S}_{i,}{S}_{j}\right)\right)$$ is the length of the common boundary of the segments S_i_ and S_j_, and T is the threshold value.

**Fig. 3:**
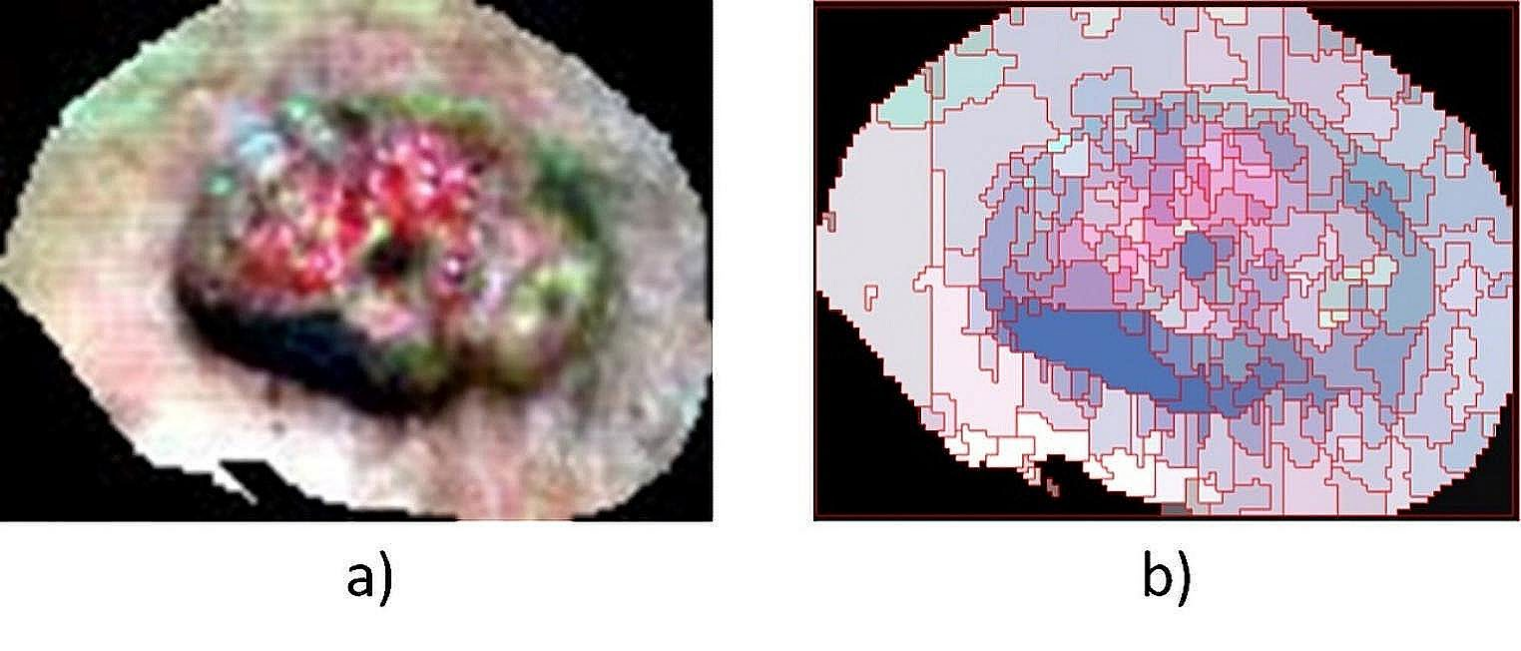
Segmentation of the hyperspectral image of an 88-year-old patient with squamous cell carcinoma of the temporal region: (**a**) original hyperspectral image (region of interest—skin tumor area); (**b**) ROI image segmented into separate segments

The end result of this two-step segmentation process is a segmentation image, where each segment is assigned the mean value of the pixels in that segment. The segmentation results using this process are dependent on two user-defined parameters, namely the scale level (SL) and the merge level (ML). The SL parameter allows the user to identify the threshold at which a certain pixel intensity value in a segment can be considered a boundary as well as the size of the segments created. The ML parameter defines the degree of similarity of adjacent segments that can be merged. In this study, several pairs of scale/merge level values were tested, and after a visual analysis of the image segmentation results by two independent physicians, the SL/ML pair of 30/75 was chosen as the appropriate one for tumor demarcation, thus allowing for a clear delineation between tumoral and normal segments, an essential element for an accurate classification of tumor areas and implicitly the delimitation of their margins based on the spectral properties of the normal and diseased skin. This step has a huge impact on the classification accuracy of skin carcinomas areas and their therapy decisions, providing an objective basis for tumor excision, improving the clearance of the tumor, and reducing unnecessary tissue destruction. A representation of a segmentated image is shown in Fig. 3.

In the third step, the classification of the individual segments resulting from the segmentation process into two classes (class 1 - tumor and class 2 - normal skin) was performed using the Spectral Angle Mapper (SAM) classification method. This classification method evaluates the degree of spectral similarity between two spectra [27, 28]. It consists in determining the spectral similarity between the reference spectra (r) and the unknown spectra of the segments (t) in the image, treating them as vectors in a space with dimensionality equal to the number of bands (n), by calculating the spectral angle between the spectra according to Eq. (3):

3 $$ \alpha ={cos}^{-1}\left(\frac{\sum _{i=1}^{n}{t}_{i}{r}_{i}}{{\left(\sum _{i=1}^{n}{t}_{i}^{2}\right)}^{1/2}{\left(\sum _{i=1}^{n}{r}_{i}^{2}\right)}^{1/2}}\right)$$

where: α is the angle between the reference spectrum and the unknown spectrum, t is the unknown spectrum, r represents reference spectrum, and n is the number of spectral bands in the hyperspectral image. The result of SAM classification is a map showing the best match of each segment to the reference samples.

**Fig. 4:**
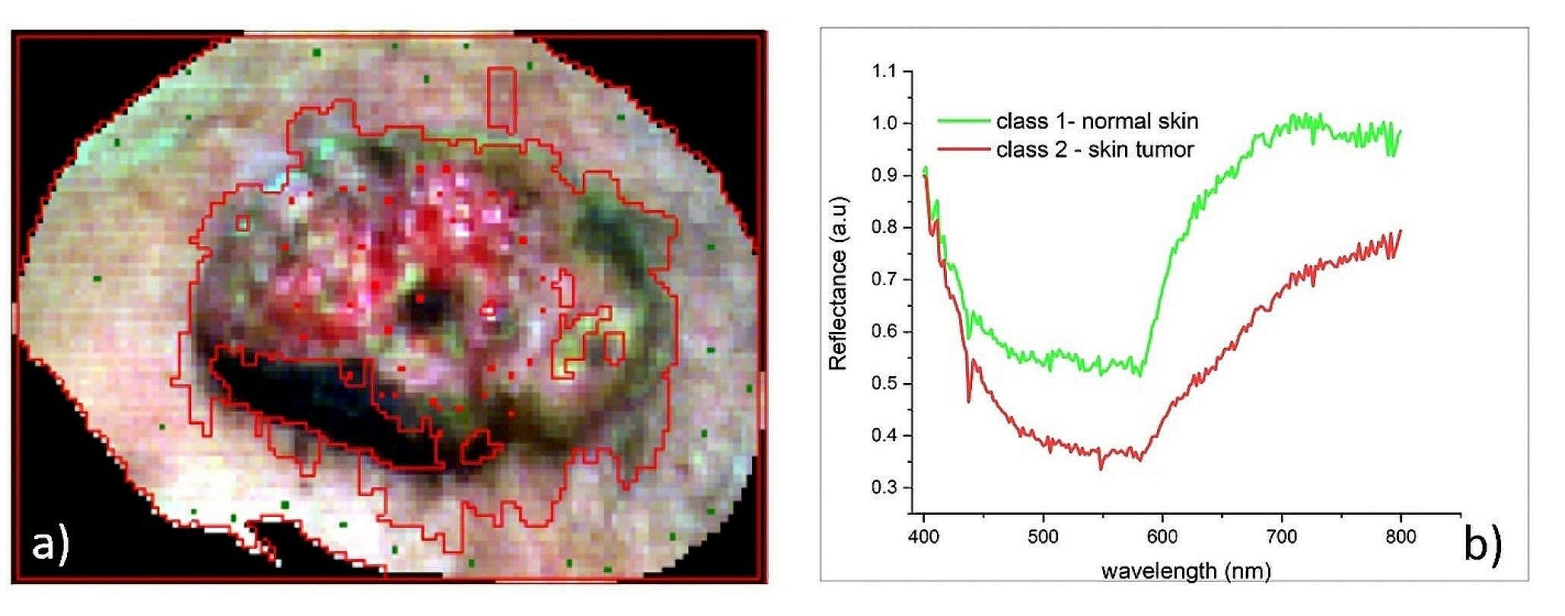
Selection of reference sample from hyperspectral image of an 88-year-old patient with squamous cell carcinoma of the temporal region: (**a**) the set of representative reference samples (training samples—green and red dots) selected by the physicians from classification vectors image; (**b**) the average reflectance spectral profile of the two tissue classes (class 1– normal skin and class 2– skin tumor)

In this study, in order to perform the SAM classification and generate the distribution maps of the two classes (tumor and normal skin) highlighting the best match of each segment to the reference samples, the average reflectance spectrum for each reference segment selected by the physicians from each segmentation image obtained from the hyperspectral data of the patients was calculated (Fig. 4).

A set of segments (∼37%) were selected from each segmentation image as reference (training) segments for each of the two classes in the hyperspectral image according to Van Niel’s rule [29] for a simple discrimination problem and the physicians’ recommendations, and the rest were used as unknown (testing) segments. A default maximum angle threshold of 0.1 radians was set to separate the spectra of the two classes (tumor and normal skin).

### Performance metric

The evaluation metric used to assess the performance of the proposed TMD method in assessing tumor margins in in vivo hyperspectral images was based on receiver operating characteristic (ROC) and the area under the ROC curve (AUC) due to its proven performance, simple calculation mode and easy manner of interpretation [30]. The ROC curve is a two-dimensional graphical representation where the true positive rate (equivalent to sensitivity) is plotted on the Y-axis versus the false positive rate (equal to 1 − specificity) which is plotted on the X-axis at various threshold settings. The area under the ROC curve (AUC) was calculated independently for all hyperspectral images in the study group as a measure of the capability of the method to distinguish between the two types of tissues (tumor/normal). The higher the values (closer to 1), the better the method performs in differentiating the tissue classes. In this study, for the calculation of the ROC curve, the threshold range between 0.10 and 0.78 (the minimum and maximum values for the spectral bands of the hyperspectral images) was considered optimal. The overall performance of the method was evaluated based on the median, minimum, and maximum AUC values.

In addition, a comparison between the TMD method and the dermoscopic method was performed. As the dermoscopic method cannot be considered as the “reference” method, the differences between the measured and calculated distances in the dermoscopic images and the TMD classification maps were compared using the statistical method proposed by Bland and Altman [31].

All analyses were performed using the statistics function of the ENVI software v 6 and SPSS software v23 (International Business Machines Corporation (IBM), New York, United States), and ORIGIN v 9.75 (2020b) (OriginLab Corporation, Northampton, United States)”.

## Results

The results consisted in classification maps that showed the limits between the two main classes of segments, which were considered tumor margins as determined with the described method.

### In vivo skin tumor margins delineation using hyperspectral imaging and the TMD method

**Fig. 5:**
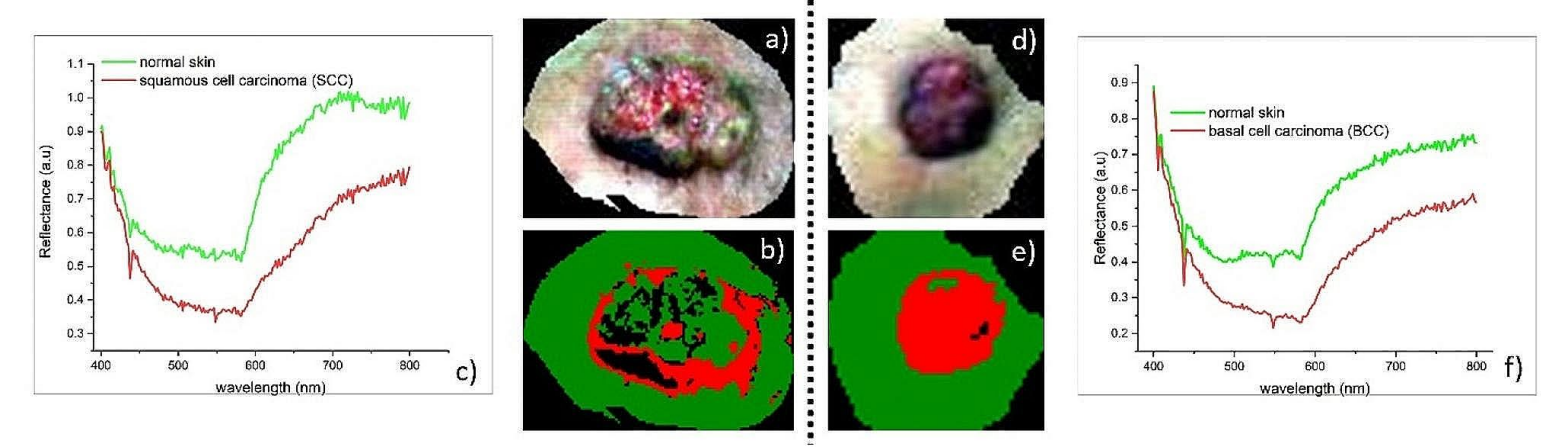
shows two representative results of the TMD method in delineating the margins of a BCC and a SCC, included in this study, from the hyperspectral images of two patients

Figure 5. Representative results of the TMD method delineating the margins of BCC and SCC. The left side of the figure shows the TMD method result on a 88-year-old man with moderately differentiated squamous cell carcinoma (pathology result) on the left temporal area. The right side of the figure shows the results of the TMD method on an 82-year-old woman with nodular basal cell carcinoma on the right temporal region. (a) and d) show the original hyperspectral images (ROI) relative only to the tumoral areas of the two patients; (b) and e) hyperspectral data classification maps showing tumor extent (red area) as determined by TMD method; (c) and f) average spectral profiles of normal and cancerous skin (SCC and BCC) used as training samples in classification process.

Figure 5b shows the SAM classification map of the segments derived from the segmentation processing based on the edge algorithm and the full lambda-schedule algorithm applied to the original hyperspectral image of the SCC tumor (Fig. 5a). A clear distribution of segments covering the SCC area (red) and those belonging to normal skin (green) can be observed. Black segments are unclassified segments with an average spectral profile different from that of the two investigated classes, segments which belong to the tumor but have a different spectral signature. This fact is probably explained by the spectral heterogeneity of the tumoral tissue, but it doesn’t alter the delimitation. This is a confirmation of the initial hypothesis made in the development of the TMD method, namely that segments with the same spectral profiles belonging to a certain area can be classified in a class. The significant differences between the spectral profiles of the two classes, both in terms of shape and intensity (i.e., ΔR = R_N_– R_SCC_ = 0.311 at λ = 700 nm) (Fig. 5c) made it possible to clearly differentiate the classes and highlight the SCC margins. A clearer delimitation was obtained in the case of patient P2 with BCC (Fig. 5e), the nodular BCC being more homogenous. Only a few segments in the tumor area were unclassified (1.6%), and the rest were classified as BCC class (25.8%) and normal skin class (72.6%). The spectral differences between the two classes (normal skin and BCC) were somehow smaller in this case (i.e., ΔR = R_N_– R_BCC_ = 0.192 at λ = 700 nm) (Fig. 5f) than in the case 1.

It is worth noting that, regardless of the patient’s normal skin type (Fitzpatrick scale), the reflectance spectrum of a tumor area (either SCC or BCC) is modified in shape and almost always reaches a value of *R* < 0.75 at 800 nm. For both types of tumors, the similarity of the shape of the average reflectance profiles (Fig. 5c and f) shows that visual diagnosis would be practically impossible, but the differences in intensity can give an answer to this challenge. These spectral similarities and differences between the segments covering the tumor area and the normal one in the image stayed at the basis of the TMD method to determine the tumor margins.

### Evaluation of the performance of the TMD method in the delineation of the tumor margin

**Fig. 6:**
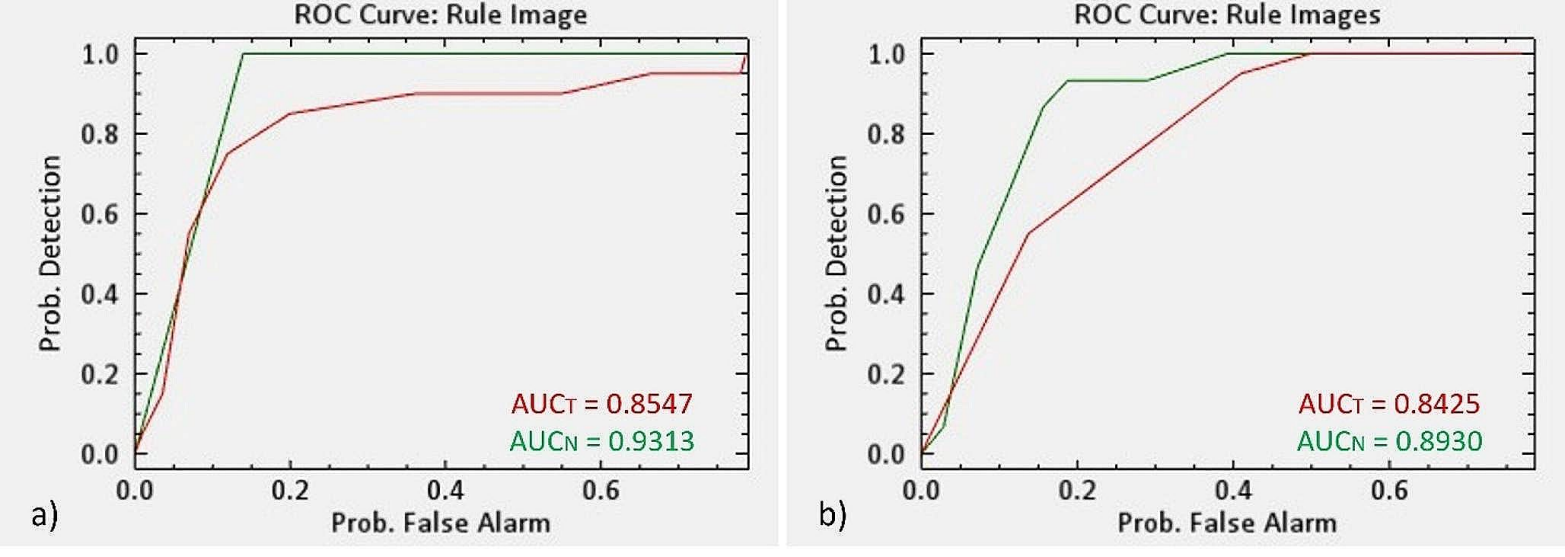
The receiver operating characteristics (ROC) curves of the TMD method. (**a**) ROC curves for patient 1 (SCC) and for individual classes: SCC (red) and normal skin (green); (**b**) ROC curves for patient 2 (BCC) and for individual classes: BCC (red) and normal skin (green)

The performance of the TMD method was assessed for each patient using receiver operating characteristic (ROC) curves. Representative ROC curves of the TMD method calculated for the above two patients and for each individual class (tumor and normal skin) are shown in Fig. 6.

It can be seen in Fig. 6 that the ROC curves for each skin tumor type and individual class are far from the bisector line, and the AUC values are relatively close to 1 (patient 1: 0.8547 (SCC tumor) and 0.9313 (normal skin), and patient 2: 0.88425 (BCC tumor) and 0.8930 (normal skin)). These results show that the TMD method performs very well in delineating the two types of skin tumors (BCC and SCC).

**Fig. 7:**
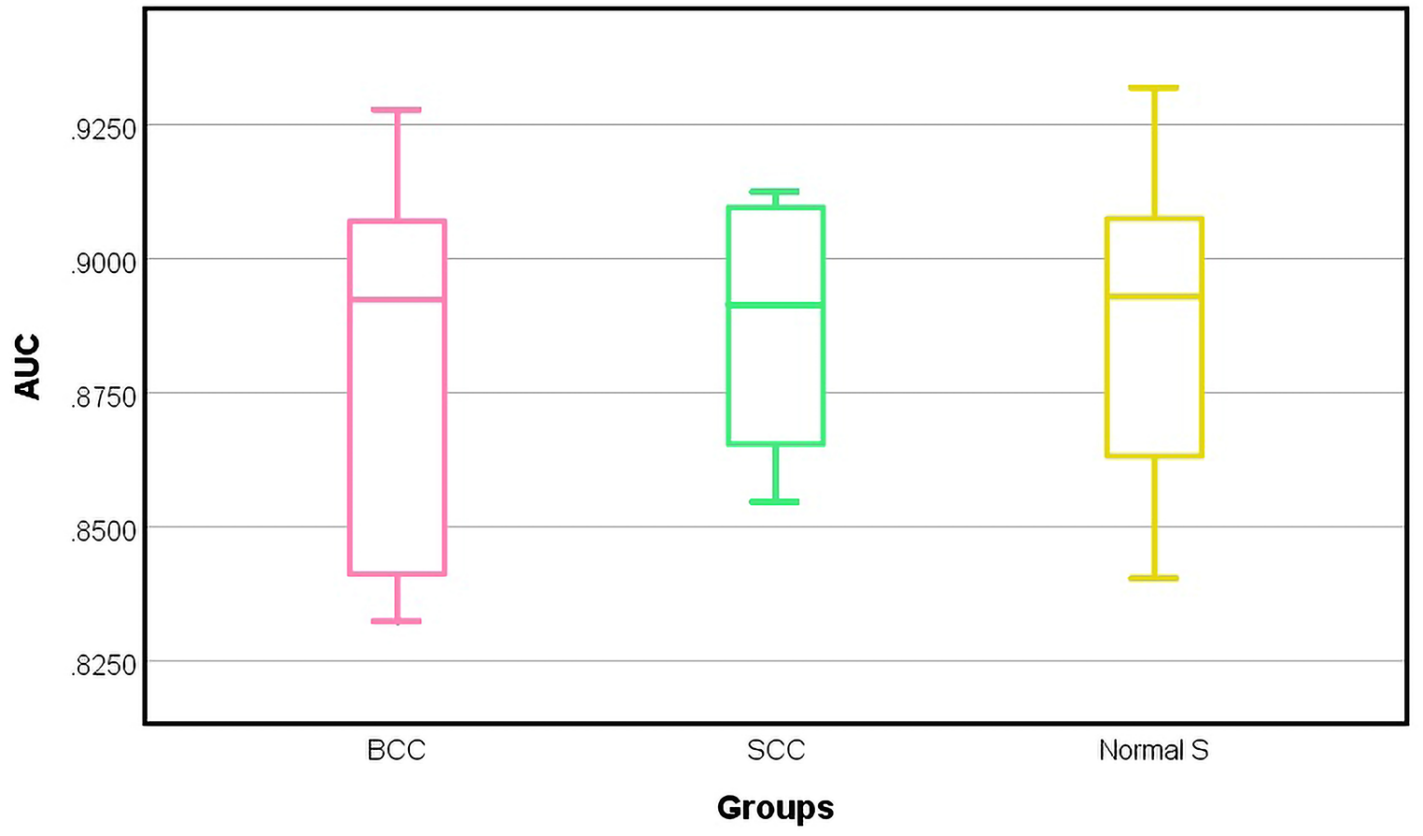
Boxplot showing the distribution of AUC values for the two types of skin cancer tumors and normal skin. The central line of each box represents the median and the edges of the box represent the upper (75%) and lower (25%) quartiles. The lower and upper whiskers denote the minimum and maximum value of AUC for each group

When the performance analysis of the TMD method based on the ROC curve was performed on the entire study group, similar results were obtained (Fig. 7).

As shown in Fig. 7, the median AUC values for each box are greater than 0.89. These results indicate that the TMD method is very efficient in delineating the types of skin tumors (BCC and SCC) from normal skin. It is also noted that the SCC box appears to have a symmetrical distribution of AUC values, with the median approximately in the middle of the box (0.8914) and the smallest interquartile range between 0.8654 and 0.9095. The minimum and maximum AUC values are 0.8547 and 0.9125. The smaller whisker distance for the SCC box and the fairly high median value demonstrates that the TMD method consistently achieves high AUC values for this group. This result is probably linked to the fact that the SCC group was more homogenous in terms of histopathological grading. On the other hand, in the BCC box the corresponding AUC values are located more towards the bottom of the box, and the interquartile range is the largest of all, ranging from 0.8399 to 0.9105. However, in this case, the maximum AUC values (0.9278) exceeded those in the SCC box, showing that some of the BCCs’ margins were better identified than for the SCCs, probably due to the particularity of nodular BCC to have clearer borders. In the classification of normal skin, the TMD method proved to have the best performance with a relatively uniform distribution of AUC values around the median value (0.8930) of the range (0.8404–0.9319) proving that normal skin is spectrally more homogenous than tumors regardless of skin phenotype.

**Fig. 8:**
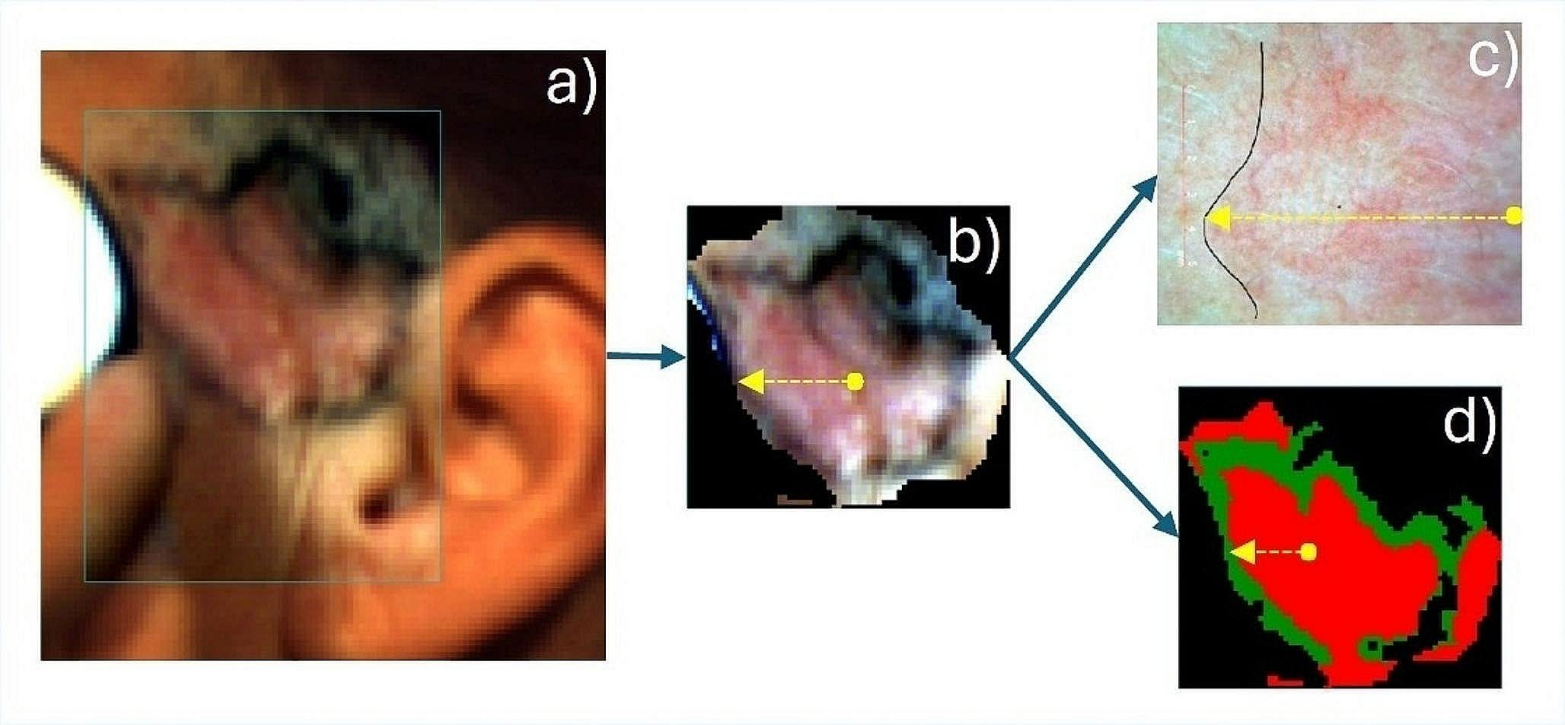
Selection of the maximum horizontal distance in tumor area for comparative analysis of the TMD method with dermoscopy: (**a**) original hyperspectral image of a basal cell carcinoma in a 57 years-old female in the temporal/preauricular region. (**b**) region of interest from the hyperspectral image with the distance drawn from the tumor center to the anterior margin. (**c**) dermoscopic image of the tumor at the anterior pole with highlighting of the tumor margin. (**d**) the classification map generated from hyperspectral data by TMD method with the maximum horizontal distance in yellow

A comparative analysis of the TMD method with the dermoscopic method was also performed to better highlight the performance of the TMD method. The dermoscopic images of each skin tumor were analyzed and the maximum horizontal distance measured from the center of the tumor to its margins (D_max−dermo_) was compared with the value of the maximum horizontal distance (D_max−TMD_) calculated from the classification maps generated from the hyperspectral data (Fig. 8).

**Fig. 9:**
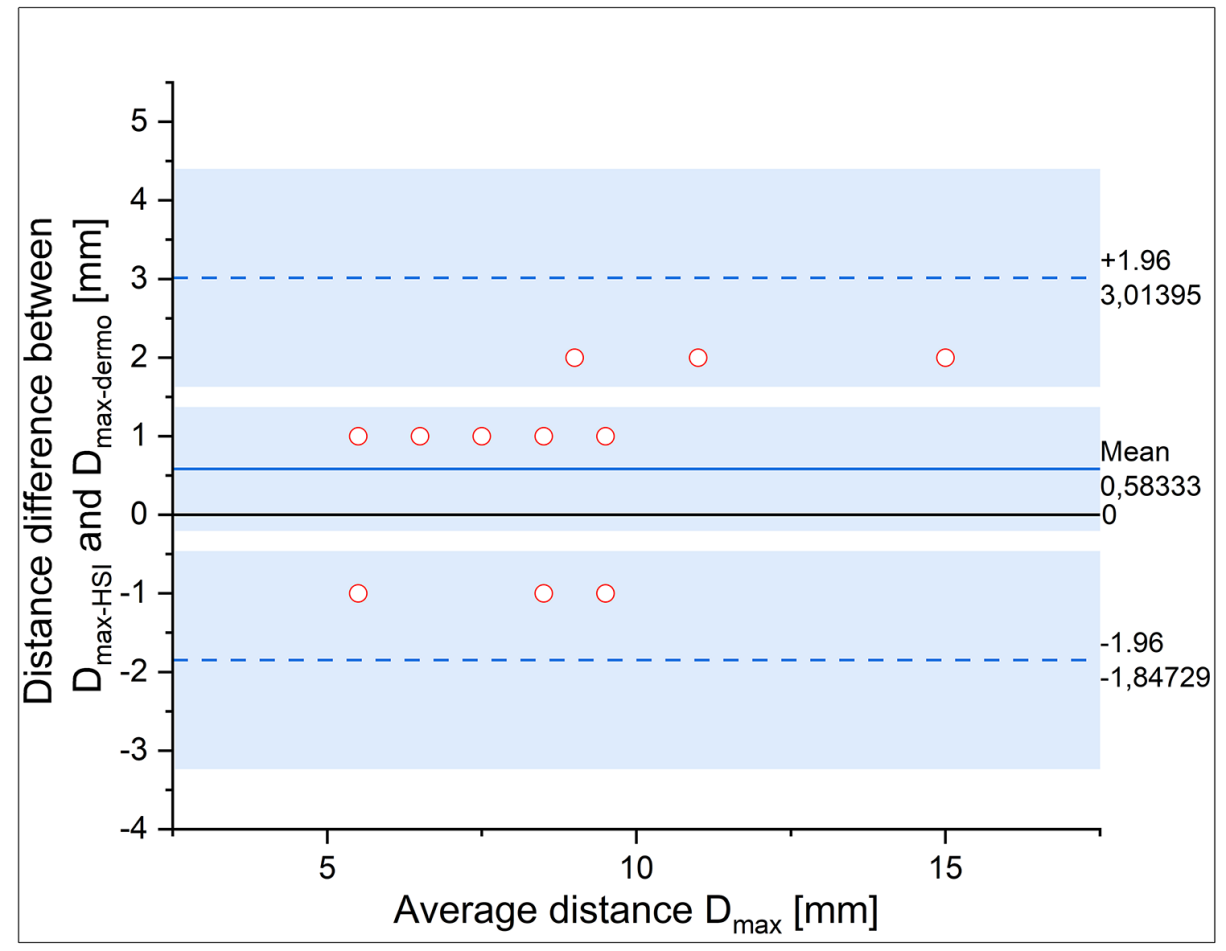
Bland-Altman plot of the difference between the distances to the margins of the tumors calculated by the TMD method and measured in dermoscopic images against the mean values. The solid blue line indicates the mean difference between TMD and dermoscopic distances. The dotted lines indicate the limits of agreement. The solid black line is the line of equality

The differences between the distances measured and calculated by the two methods (dermatoscopy and the TMD method) against the mean values according to the Bland and Altman method [31] are shown in Fig. 9.

Visual examination of the Bland-Altman plot reveals that the mean difference in distances is 0.58 mm and the line of equality (line corresponding to a zero differences) lies within the confidence interval of the mean difference with non-significant bias. This graph also shows that, in most cases, the distance calculated by the TMD method is greater than that measured by dermoscopy. This fact might be explained by the amount of spectral information that the TMB method provides in comparison with dermoscopy when it comes to establishing tumor margins. The agreement limits are narrow enough (between − 1.85 and 3.01 mm) and there is no estimation error even if the number of cases is relatively small. Therefore, there is no statistically significant difference between the two methods, but with a slight tendency of TMD to detect wider tumor extension than dermoscopy.

## Discussion

Our article proposes a new hyperspectral imaging-based approach for in vivo delineation of carcinomas of the face (both BCC and SCC). The method is based on the fact that carcinomas and normal skin have different spectral signatures due to complex morphological differences between the two types of tissue. During the analysis of hyperspectral images of tumors and surrounding normal skin, segmentation was a decisive step, because it provided simpler maps with less data to be analyzed by the classifier. Some researchers have used pixel-based analysis methods for tumor delineation from hyperspectral images [21, 23], which are of high complexity and time consuming. By grouping pixels with similar features into a single group through an image segmentation process and performing further group analysis, as we proposed, the computational complexity is greatly reduced. Moreover, as long as our goal was to define only the margins of the tumors, this approach proved to be sound and effective. That explains the fact that, in some maps (like the one in Fig. 5b), some segments in the center of the tumor remained unclassified.

Several studies involving hyperspectral imaging in tumor margin detection were done, the majority being ex vivo (on surgical specimens) [14–18]. Surgical specimens have lower concentrations of one of the main chromophores (hemoglobin), which significantly influence spectral signatures, making comparison with in vivo images difficult. Fewer studies were performed in vivo. Two of them [21, 22] had as subject pigmented lesions (lentigo maligna and lentigo maligna melanoma) and good results were reported by both groups. In these cases, the presence of melanin as a predominant chromophore allowed for an easier detection of tumor extension with less complex analysis of the hyperspectral images (linear mixture model for vortex component analysis and filter vector algorithm [21], or diffusion maps technique [22]. An interesting in vivo approach was described by Zhang et al. [11] using diffuse reflectance spectroscopy, which, by using selected physiological parameters, could identify normal or tumoral tissue in the point where the probe was placed. Despite being practical, the method cannot provide comprehensive maps due to its inherent limitations. The only hyperspectral image-based approach performed in vivo on BCCs reported so far belongs to Salmivuori et al. [23]. They used the same linear mixture model analysis method as in [21] with 4 mathematical model variations, but the interpretation of the abundance maps (choosing the endmembers) was done by humans in every case, a fact that introduces a certain amount of subjectivity. Our machine learning-based approach is making the method more reliable and repeatable in a more objective way. Salmivuori’s study has as main advantage that they succeeded in using histopathological results as ground truth, even though it was done only in certain selected areas of the image. In our study, a comparison with dermoscopic findings was used in order to provide proof about the method’s reliability. Although there was no statistical difference between the two methods, in most cases TMD method tended to provide larger tumor margins than dermoscopic evaluation.

The presented method has important elements of originality (like the approach using segments instead of pixels) and it seems easy to use with the trained SAM and less involvement of human decisions. It may become a valuable tool in preoperative planning for carcinoma resection in functional or esthetic areas like the face, helping the surgeon to reduce the rate of incomplete removal of the tumor while preserving as much as possible the healthy skin or deeper structures Moreover, it may also prove to be a valuable tool for monitoring the effectiveness of future developments in cancer treatment, including new nano polymers which could improve the overall clearance rate [32].

Study has its limitations, but many of them can be overcome in further studies. The low number of subjects (tumors), and the heterogeneity in terms of histological subtypes are the main ones. This is the reason the authors named it a preliminary study. A comparison with the golden standard of histological examination would have brought more value. This is a difficult task, though, as long as, once transformed into a surgical specimen, the tissues shrink, and spatial relations are modified. Solving this issue would be the next needed step for method validation.

## Conclusions

In conclusion, our study proved that in vivo hyperspectral imaging combined with segmentation and classification processes based on machine learning algorithms can become an objective aid in preoperative assessment of carcinoma margins irrespective of their histopathological type and can provide valuable information regarding the extent of safe tumor excision.
